# An inverse planned oil release validation method for estimating oil slick thickness from thermal contrast remote sensing by in-scene calibration

**DOI:** 10.1016/j.mex.2022.101756

**Published:** 2022-06-16

**Authors:** Ira Leifer, Christopher Melton, William J. Daniel, Jae Deok Kim, Charlotte Marston

**Affiliations:** Bubbleology Research International, Inc., Solvang CA 93463, USA

**Keywords:** Floating oil, Airborne oil thickness mapping, Emissivity, Oil collection

## Abstract

This study demonstrates a method to estimate floating oil slick thickness based on remote sensing of thermal infrared contrast. The approach was demonstrated for thick oil slicks from natural seeps in the Coal Oil Point seep field, offshore southern California. Airborne thermal infrared and visible spectrum remote sensing imagery were acquired along with position and orientation data by the SeaSpires™ science package. Remote sensing data were acquired in the cross-slick direction of oil slick segments that were targeted for collection, termed “collects.” A collect consisted of booming, skimming, and offloading the oil slick segment into buckets for analysis at the laboratory. Each collect provided an *in-scene* calibration point of oil thickness versus brightness temperature contrast, *ΔT_B_*, where *T_B_* is the sensor-reported temperature based on the emitted thermal radiation and differs from the true temperature due to the oil's emissivity. *ΔT_B_* is the *T_B_* difference between the oil and oil-free sea surface. Thus, this study is a reverse planned oil-release experiment that demonstrates the value of natural seeps for oil spill science.

• *Novel approach to quantify floating oil thickness*

• *Custom modified weir skimmer used with added floor and structural strengthening*

## Graphical abstract

**Figure:**
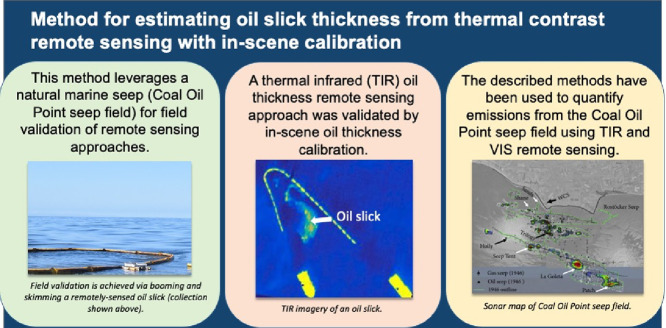

## Nomenclature

Term Units: Definition
Dem -: digital Elevation Model
Inu -: inertial Navigation Unit
Pdf -: probability Distribution Function
Raid -: redundant Array of Inexpensive Disks
Rgb -: red Green Blue
Rms -: root Mean Square
Rpm -: rotation Per Minute
Sas -: seep Assessment Study
Tir -: thermal InfraRed
Uv-nir -: ultraviolet to near-infrared
Vis -: visible Spectrum
$T_{BP\_O}$ °C: peak temperature of fit to *Φ_o_*(*T_B_*)
$T_{BP\_W}$ °C: peak temperature of fit to *Φ_W_*(*T_B_*)
$T_{BP\_W1}$ (°C): peak temperature of fit to *Φ_W1_*(*T_B_*)
*a_o_* (-): amplitude of *Φ_o_*(*T_B_*)
*a_W_* (-): amplitude of *Φ_W_*(*T_B_*)
*a_W1_* (-): amplitude of *Φ_W1_*(*T_B_*)
*b, c, d* -: fit parameters
*f_1_* (°C): gap-filling transition function, part 1
*f_2_* (°C): gap-filling transition function, part 2
*FD* m: first difference
*h* mm: oil layer thickness
*h(ΔT)*: calibration function
*I_n_* -: horizontal image coordinates of pixel *n*
*J_n_* -: vertical image coordinates of pixel *n*
*n* -: reference pixel number
*R^2^* -: correlation Coefficient
*T* °C: temperature
*T_BO_* °C: oil brightness temperature
*T_BW_* °C: water brightness temperature
*T_BW_*(*y*) °C: cross slick *T_B_* profile
*x* m: *along slick coordinate*
*y* m: *cross slick coordinate*
*y*_1_ m: distance parameter in gap-filling transition function
*y*_2_ m: distance parameter in gap-filling transition function
*Z* m: airplane altitude
*σ* -: roll
*γ* -: yaw
*Δlat* m: latitude offset from pitch + roll
*Δlat_1_* m: latitude offset from roll
*Δlat_2_* m: latitude offset from pitch
*Δlon* m: longitude offset from pitch + roll
*Δlon_1_* m: longitude offset from roll
*Δlon_2_* m: longitude offset from pitch
*ΔT* °C: temperature contrast with oil-free water
*ΔT_B_* °C: brightness temperature contrast with oil-free water
Φ -: pixel *T_B_* probability distribution
*Φ_o_* -: oil *T_B_* probability distribution
*Φ_W_* -: seawater *T_B_* probability distribution
*Φ_W1_* -: wake water *T_B_* probability distribution
*δ* -: pitch

## Specifications table

**Table:** 

Subject Area:	Environmental Science
More specific subject area:	*Oil Pollution*
Method name:	*Floating Oil Thickness from Thermal Infrared Remote Sensing*
Name and reference of original method:	*n/a.*
Resource availability:	*N/A*

## Method details

### Overview

The approach combines sea surface and airborne measurements as part of the Seep Assessment Study (SAS), described in Leifer et al. [1]. The study collected thermal infrared (TIR) and visible (VIS) spectrum imagery, as well as viewing position and orientation data of a targeted oil slick segment by the oil spill mapping science package, SeaSpires™. The targeted oil slick segment was boomed, skimmed, collected, and weighed in the laboratory. These two datasets (remote sensing and surface) provided an *in-scene* calibration of oil thickness to remote sensing data. In essence, the *in-scene* calibration is a reverse planned-release experiment.

#### Oil slick remote sensing

Currently, oil slick thickness remote sensing is at an early development stage. Specifically, Fingas [2] notes, “There are presently few reliable methods, either in the laboratory or in the field, for accurately measuring oil-on-water slick thickness.” The most common approach to remote sensing oil is by observation of reflected visible spectrum sunlight due to the commercial availability of low cost and low weight video cameras [3]. The thickness of oil by VIS appearance is codified in the Bonn Agreement [4] and the ASTM Standard [5]. Due to using reflected solar radiation, VIS remote sensing is limited to clear (cloud-free) daytime skies [6] and high solar angles [3]. Many factors complicate the VIS oil slick appearance, including viewing angle, atmospheric interferences, oil type, sub-pixel heterogeneity, seawater color (due to dissolved organic materials), and sea state [2]. As such, Fingas [2] notes the only definitive visible thickness range is for rainbow appearance slicks, which are 0.3–5 µm.

An alternate, commercially available, spectral approach uses microwave brightness. Microwave brightness varies with thickness; however, it does so in a cyclical manner with several thicknesses yielding the same microwave brightness. In principle, systems with multiple microwave wavelengths can address the non-uniqueness of the derived thicknesses [7], although Skou [8] found different thicknesses for the different wavelengths. Additionally, system resolutions are order tens of meters, implying a significant spatial averaging of a non-linear response. Oil slicks thicker than rainbow slicks (>50 µm thick) typically exist in the environment as reddish-brown oil-water emulsions – microdroplet mixtures [4]. Emulsions affect microwave scattering in the oil layer [9], complicating thickness derivation. Emulsions also affect the visible appearance.

#### Oil slick thicknesses

Very thin slicks, often termed sheens (0.01–0.3 µm), appear silvery in the VIS due to reflecting the sky color [4]. Rainbow appearance slicks are one to several integer multiples of the wavelength of light, with the colors arising from thin film effects [10]. Slicks thicker than rainbow appear metallic due to combining reflected sky color with the oil's true color. Slicks appear metallic for thicknesses from approximately 5 to 50 µm. Slicks thicker than 50 µm show the oil's true color.

Given that most (∼90%) of the volume of an oil slick is in the thick (true color) portions of the slick [11], the Bonn Agreement provides little guidance for assessing the thickness or volume of the thicker, actionable oil slick areas. This study uses a different definition of thick oil - actionable, corresponding to oil thicker than 0.5 to 3 mm. This actionable threshold varies with weathering, mitigation approach, and other factors [2,12]. This thick oil definition based on actionable overlaps with the oil thickness definition based on thermal processes in oil slicks. Note, actionable oil is significantly thicker than the thickness ranges addressed by visible and microwave approaches.

In the visible spectrum there is an absence of petroleum hydrocarbon diagnostic spectral features. A diagnostic spectral feature in the case of oil is unique to petroleum hydrocarbons and can discriminate oil from lookalikes, such as biofilms. Thus, distinguishing oil slicks from lookalikes relies on non-spectral approaches. One approach is to analyze the slick spatial patterns for consistency with expected spatial patterns for oil [3]. Alternatively, multiple different remote sensing approaches can be combined, e.g., radar, thermal, ultraviolet, etc. [1,^13^,14] – different spectral regimes exhibit different lookalike signatures. Another approach considers ancillary data such as winds [15].

Quantitative oil thickness can be derived using near-infrared diagnostic spectral features around 1.6 µm and 2.3 µm. These features were used during the Deepwater Horizon (DWH) oil spill by the AVIRIS (Airborne Visual Infrared Imaging Spectrometer) hyperspectral imager [16,17] and retrospectively on the DWH AVIRIS data [18]. Specifically, the skew in the spectral feature allows derivation of the slick thickness. This skew arises because longer wavelength light penetrates the oil better (less absorption in the oil) than shorter wavelengths and thus is absorbed more strongly by the underlying water. This wavelength-dependent absorption alters the spectral feature's shape.

The approach also yields the pixel's fractional water / oil coverage and emulsion level, both of which affect the spectral feature. Emulsions scatter light with a wavelength dependency that significantly alters further the spectral feature's shape. Fortunately, the spectral feature is weakly sensitive to emulsion droplet size [18]. To date, near-infrared hyperspectral oil thickness remote sensing has not been field validated for oil slicks of known thickness. Furthermore, the effect of sub-pixel thickness and emulsion heterogeneity on the derived thickness remains unknown. Currently, commercial near-infrared imaging spectrometers are unavailable.

#### Thermal infrared oil slick remote sensing

A commercially-accessible approach with significant promise is TIR remote sensing. TIR remote sensing uses emitted thermal radiation instead of reflected solar radiation, enabling daytime and nighttime operations and even under cloudy skies. TIR cameras provide the brightness temperature from the remotely-sensed emitted radiation. The Stephan Boltzman Law relates the emitted radiation to the surface temperature [19] and is,

(1) $$E_{B}=\varepsilon \sigma T_{S}^{4}$$

where *E_B_* is the blackbody emissive power, *σ* is the Stefan-Boltzmann constant, *ε* is the emissivity, and *T_S_* is temperature, which differs from the brightness temperature, *T_B_*, which is the apparent temperature, and is,

(2) $$T_{B}=\varepsilon T_{S}.$$

Oil slick TIR remote sensing leverages the differential heating of oil compared to the surrounding oil-free water, with thicker oil warming more than thin oil [20], [21], [22], [23], [24]. Many factors contribute to the temperature contrast including the oil-water difference in emissivity, heat capacity, lower albedo, thermal conductivity, among other physical, chemical, and optical properties [23,^25^,26].

Leifer et al. [1] describes the underlying theory. In brief, the oil slick absorbs a portion of the downwelling radiation. Thus, with increasing thickness, oil heating increases until saturation at several optical depths. The absorbed energy then heat transfers to the cooler, underlying water and, in the case of thick oil, to the overlying air. Heat also flows from the warmer air to the cooler water – typical conditions for strong daytime solar insolation. Heat transfers across the oil's air and water boundary layers into the bulk media. Also, radiation is emitted (lost) from the upper air-oil interface. The thermal structure (and thus surface brightness temperature) depends on these radiative absorption and heat transfer processes and the oil's optical and physical characteristics.

#### Study motivation

Herein, we present the SAS methodology. The SAS is described in Leifer et al. [1] and investigated TIR oil slick thickness remote sensing. The SAS was applied to the perennial and continuous thick oil slicks from the Coal Oil Point seep field. These slicks provide an opportunity to field validate oil slick remote sensing. This opportunity is particularly important, given that planned releases are infeasible in US waters, and the priority during an oil spill is mitigation, not science. As a result, field validation of oil spill remote sensing approaches largely has been lacking [2,3]. Furthermore, permitting is unnecessary for a natural seep oil slick, there are no spill startup dynamics, and experimental repetition under diverse sea state and meteorology conditions are feasible [27].

The approach involved collecting an oil slick segment that also is remotely sensed. In essence, the SAS is a reverse planned release experiment - the remotely sensed oil is boomed, skimmed, collected, and then assessed in the laboratory. In a planned release, a quantity of oil is released into the environment and then remote sensed.

The SAS targeted thick, actionable oil slicks with the definition of thick versus thin oil based on the underlying heat and radiative processes. Leifer et al. [1] found that for Coal Oil Point oil, the transition from thin to thick oil based on the optical and thermal characteristics was 500–1000 µm. This definition differs from the Bonn Agreement, which is based on visible appearance. This physics-based definition overlaps with an operational definition of thick oil – many mitigation approaches, such as *in situ* burning, only work on thick oil [2,12].

### Detailed approach

#### Overview

**Fig. 1:**
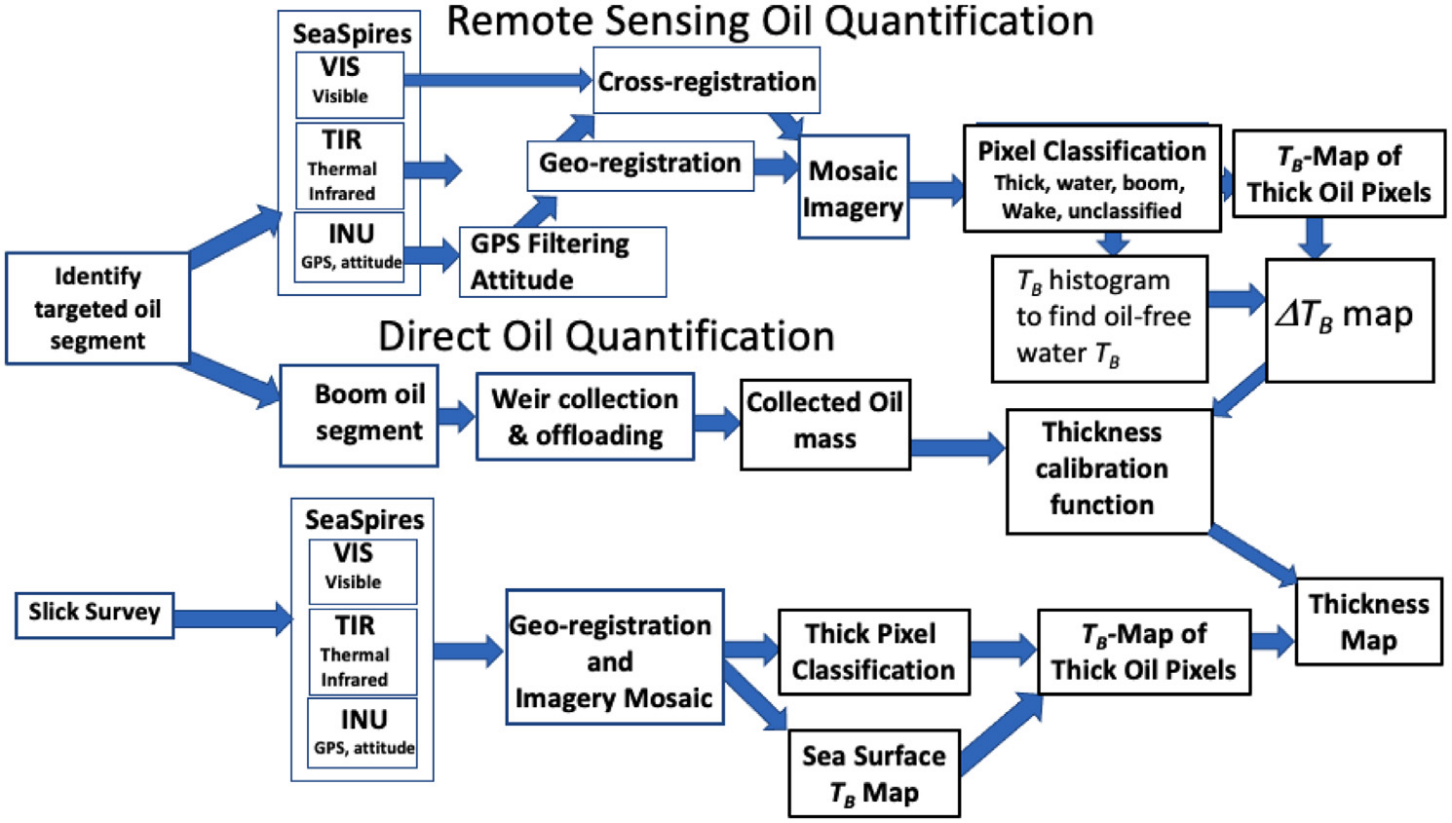
The Seep Assessment Study flowchart. *T_B_* is brightness temperature, *ΔT_B_* is the brightness temperature contrast.

The SAS approach is shown schematically in Fig. 1 and has two components. 1. Oil thickness quantification by airborne remote sensing. 2. Surface oil collection and volumetric quantification to derive oil thickness. The first step is to identify a target oil segment and line up both platforms – the vessels to boom the segment and the airplane to repeatedly image the segment while it is being collected. Once the oil boom has cut the oil slick segment, the boom vessels drift forward until the oil slick segment accumulates at the boom apex. Then the collected oil is offloaded from a modified weir skimmer – either by a pump or manually by a pool skimmer net into 5-gallon buckets. The buckets are sealed and returned to the laboratory for evaluation. Specifically, the mass and volume of the recovered oil and water and the emulsion level by centrifuging.

Airborne data are geo-registered based on INU (inertial navigation unit) position and attitude data. The INU data are filtered for timebase irregularities and then used to project TIR imagery onto a uniform UTM grid, i.e., geo-registration. Geo-registered TIR imagery then are photo-mosaicked to cover the areas of slick interest. Then, the visible imagery is cross-registered to the TIR imagery using a digital elevation model (DEM) to account for differences in orientation between the TIR and VIS cameras. This provides a companion VIS photomosaic to the TIR photomosaic. A semi-supervised approach classifies scene element pixels in the TIR and VIS imagery. Scene elements include boat and oil boom, boat wake, oil-free water, thick oil, thin oil, and unclassified. From a practical point of view, unclassified pixels were either thin oil or oil-free water.

Next, the *T_B_* contrast, *ΔT_B_*, is determined. *ΔT_B_* is the difference between the oil *T_B_* and *T_B_* for oil-free water and is averaged over the area of thick oil. This *ΔT_B_* is compared to the slick thickness, *h*, which is mass (kg) / [area (m^2^) * density (kg/m^3^)]. The calibration function is derived by fitting *ΔT_B_* to *h*. This calibration function is applied to *ΔT_B_* maps to create an oil thickness map. For slick surveys, the oil-free surface temperature is modeled as a piecewise polynomial function across the oil slick.

#### SeaSpires oil spill mapping science package

**Fig. 2:**
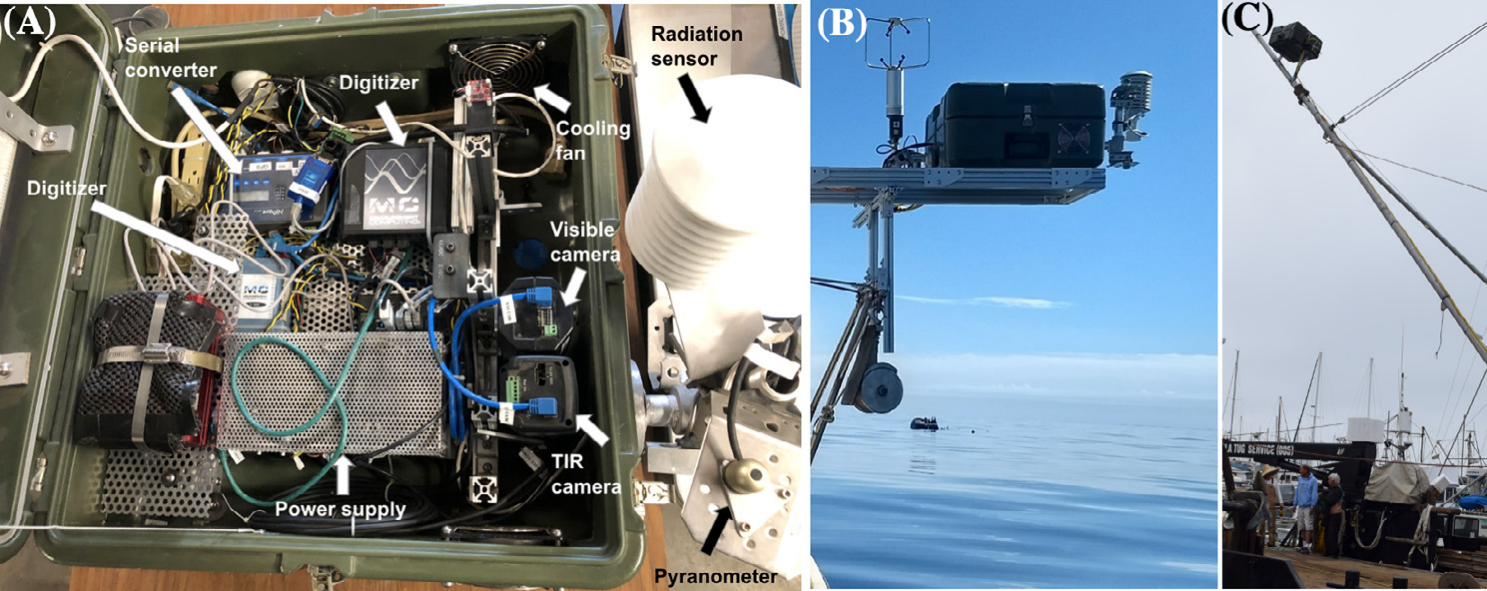
(**A)** SeaSpires interior, components labeled on figure. (**B)** SeaSpires installed off the cabin of a low-profile boat, (**C)** SeaSpires installed on a boom arm at 10-m above the water.

**Fig. 3:**
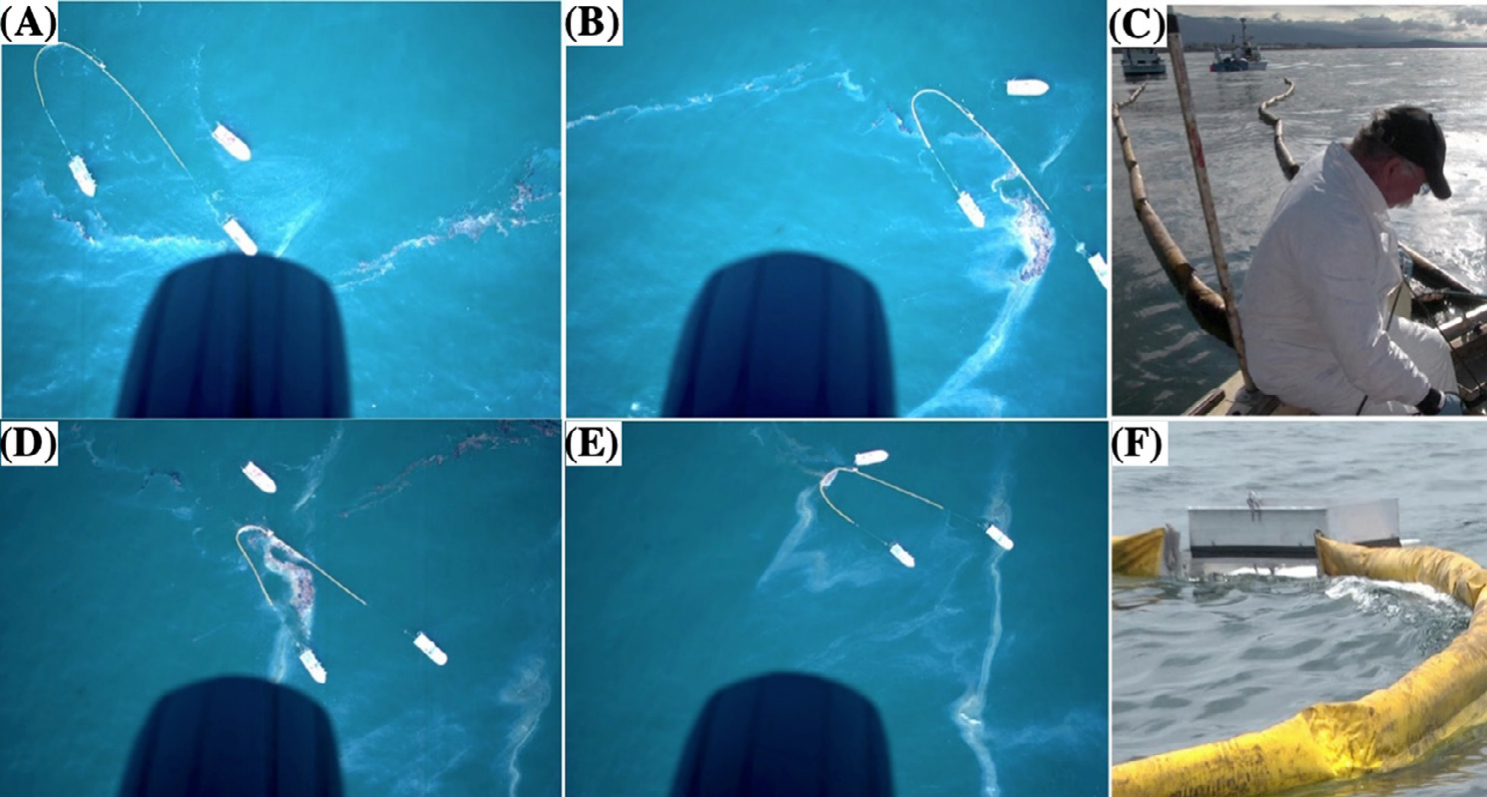
**(A)–(D)** Airplane image sequence showing boom oil collection of an oil slick segment on 23 May 2016. (**E)** Oil offloading operation. (**F)** Asymmetric boom-skimmer deployment.

The SeaSpires™ oil spill mapping science package (Fig. 2) acquired remote sensing data for the SAS (Fig. 3A–D). SeaSpires supports two VIS video cameras and one TIR video camera. The visible cameras are a high-resolution 30-megapixel video camera (7 K HDPro, Avigilon, TX), denoted HDPro, and a wide-angle 1-megapixel video camera (1.0MP-HD-DN, Avigilon, TX), which provided flight-targeting guidance. A research-grade thermal infrared (TIR) camera (A655sc, FLIR, NH), denoted A655, recorded TIR video. TIR images were 480 × 640 pixels. Although closely aligned, video image analysis revealed a slight angular offset between the 7 K and A655sc cameras. Video and control signals route into a portable computer with an attached RAID array for monitoring and recording video and other data.

Additional SeaSpires instruments include an upwards and downwards UV-NIR spectrometers (HR4000 Ocean Optics), downwelling and upwelling visible sensors, and TIR radiation sensors matched to the TIR camera and spectrometers (Apogee Instruments). Time and position are provided by a GPS time server (Time Machine 1000A) and a GPS (19x, Garmin). SeaSpires is configured for installation in the wheel well of a Cessna 2015 airplane for airborne deployment. An INU (VN-300, VectorNav, Dallas, TX) provides location and orientation data. Video cameras are nadir mounted and view the sea surface through ports in a customized cowl that prevented wind vibrations.

The INU was mounted to the airplane cockpit dashboard and used two high sensitivity GPS receivers and antennas and MEMS (Micro Electro Mechanical Systems) inertial sensors. The antennae were mounted approximately 1-m apart with good sky visibility. Static RMS (root mean square) accuracies are 0.3° heading and 0.5° pitch roll with dynamic accuracies of 0.1° and angular repeatability of 0.1° The INU provides orientation data at 50 Hz and GPS data at 5 Hz.

For boat deployments, SeaSpires measures meteorology (3D sonic anemometer, humidity, temperature), which are connected by serial servers (5450, NPort, Moxa, Taiwan) to the portable computer (HP spectre 360) for recording, based on an asynchronous streaming approach described in Leifer and Melton [28].

The SAS was tested on thick oil slicks from the Coal Oil Point natural marine hydrocarbon seep field, offshore Southern California [29]. Seepage from the COP seep field escapes from several square kilometers of the seabed as oily and non-oily bubbles and bubble plumes [27,30]. Oil emissions were estimated at 100 bbl day^−1^ in 1995 [31].

### Oil collection

Two 33-m long, 12.5 cm (6″) diameter harbor booms were connected by two ∼10-m tow ropes to two fishing boats on one end and to both sides of a modified weir skimmer (Fig. 3F). Skimmer modifications included additional buoyancy, splash guards, and structural strengthening. The modified skimmer successfully collected oil in seas up to 2 m and wind speeds up to 11 m s^−1^ (scattered rolling whitecaps). An additional important modification was a latchable door to prevent oil loss during booming. Specifically, for an open door, some of the boomed oil entered the weir where it became heavily emulsified by waves that also entered the skimmer weir. This heavily emulsified oil tended to sink immediately if it escaped the skimmer. The weir skimmer door is closed during booming to prevent oil from entering the skimmer before the offloading vessel had moved into position.

**Fig. 4:**
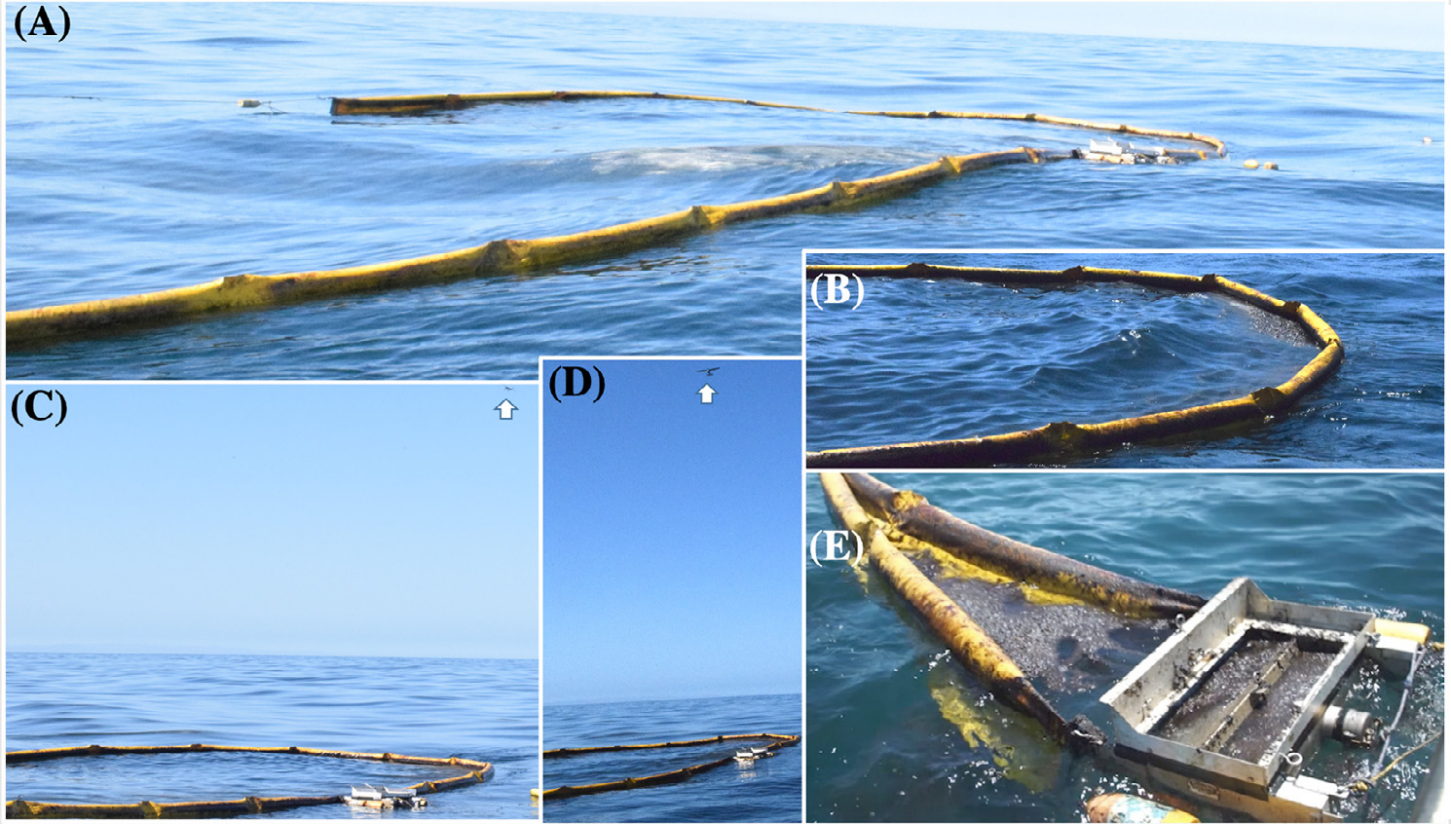
**(A)** Beginning a collect of an oil slick segment. (**B)** Evening the oil boom. (**C)** Airplane lining up a collect acquisition. (**D)** Airplane lined up for collect remote sensing acquisition. (**E)** Collapsing the boom for offloading. Photos on 23 May 2016.

When starting a collect, the two boom vessels motor very slowly towards a targeted oil streamer segment, guided by communication with the airplane (Fig. 4). The boom is towed asymmetrically, offsetting the skimmer from the boom apex (Fig. 4A). Motoring is very gentle – steady and in a nearly straight line – to avoid disrupting the oil slick and possibly inducing oil entrainment (sinking). A third offload vessel maneuvers along the boom and provides close observations for communication to the boom vessels while confirming that oil is not entrained into the water column from the boom. Continuous communication was needed to coordinate engaging and disengaging boat motors to ensure the drift is slow and smooth through the oil slick and then to allow it to drift largely undisturbed by the boom legs to the boom apex. Then, the boom mouth was closed and the trailing boom vessel moved forward to make the booms symmetric – shifting the skimmer to the boom apex (Fig. 4B). Closing the boom and equalizing the boom legs rolled the oil at the boom apex, which incorporated air into the oil slick, stabilizing the slick against entrainment. The offload vessel moved into position and opened the skimmer door (Fig. 4E) and a throttled, 160-gallon min^−1^ gasoline-powered water pump is attached to the skimmer port, which is located in the skimmer's hull. The oil boom is opened slightly and pumping offloads the oil. Oil that was too thick to flow to the offload port was offloaded with a pool leaf skimmer. Off-loaded oil was stored in 20-liter (5 gallon) plastic buckets whose lids were sealed and labeled with time, collect number, and fill order. Collection efficiency (negligible loss) was confirmed through careful monitoring by the sea surface observers and later inspection of aerial imagery. The approach allowed oil collection in seas of up to 2 m and wind speeds up to 11 m s^−1^ (rolling whitecaps).

**Fig. 5:**
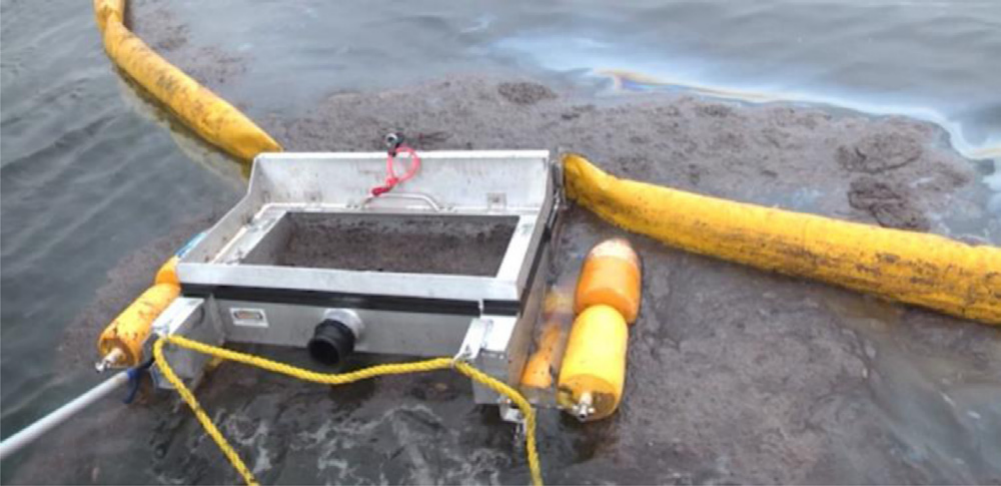
Photo of skimmer oil leakage – a failed collect.

Occasionally oil leaked from the skimmer (Fig. 5), causing the collect to be abandoned – a process where one boat released its side of the boom and the other boat moved rapidly forward with the boom streaming behind. This boat then proceeded to a new slick segment for the next collect attempt, where the boom's free side was re-attached to the second boom vessel.

Each bucket was weighed back at the laboratory (NTEP Ranger 7000, Ohaus Corp., NJ, 35 kg, 0.5 g accuracy; LC3200D, Sartorius, Germany, 3 kg, 1 mg accuracy), referenced with Class 1 calibration weights (Troemner, NJ). The analysis primarily focused on collects that were dominated by manually skimmed oil (estimated >97%) with negligible water in the buckets. Lab analysis determined the fraction of water in the oil by subsampling and centrifugation for at least 1 h at 3000 RPM. Centrifugation separates the oil and water in the emulsion allowing quantification of the level of emulsification by measurement of the total sample volume and the water volume.

### Remote sensing analysis

#### Imagery geo-registration

Geo-registration used the IMage GeoRectification And Feature Tracking (ImGRAFT) toolbox in MATLAB (2016a, Mathworks, MA), described below. In brief, images are projected onto a UTM grid and re-gridded to 0.2-m resolution using 2D cubic-spline interpolation. Post-processing of INU data yielded an uncertainty of approximately 0.02 m, significantly less than the grid resolution.

**Fig. 6:**
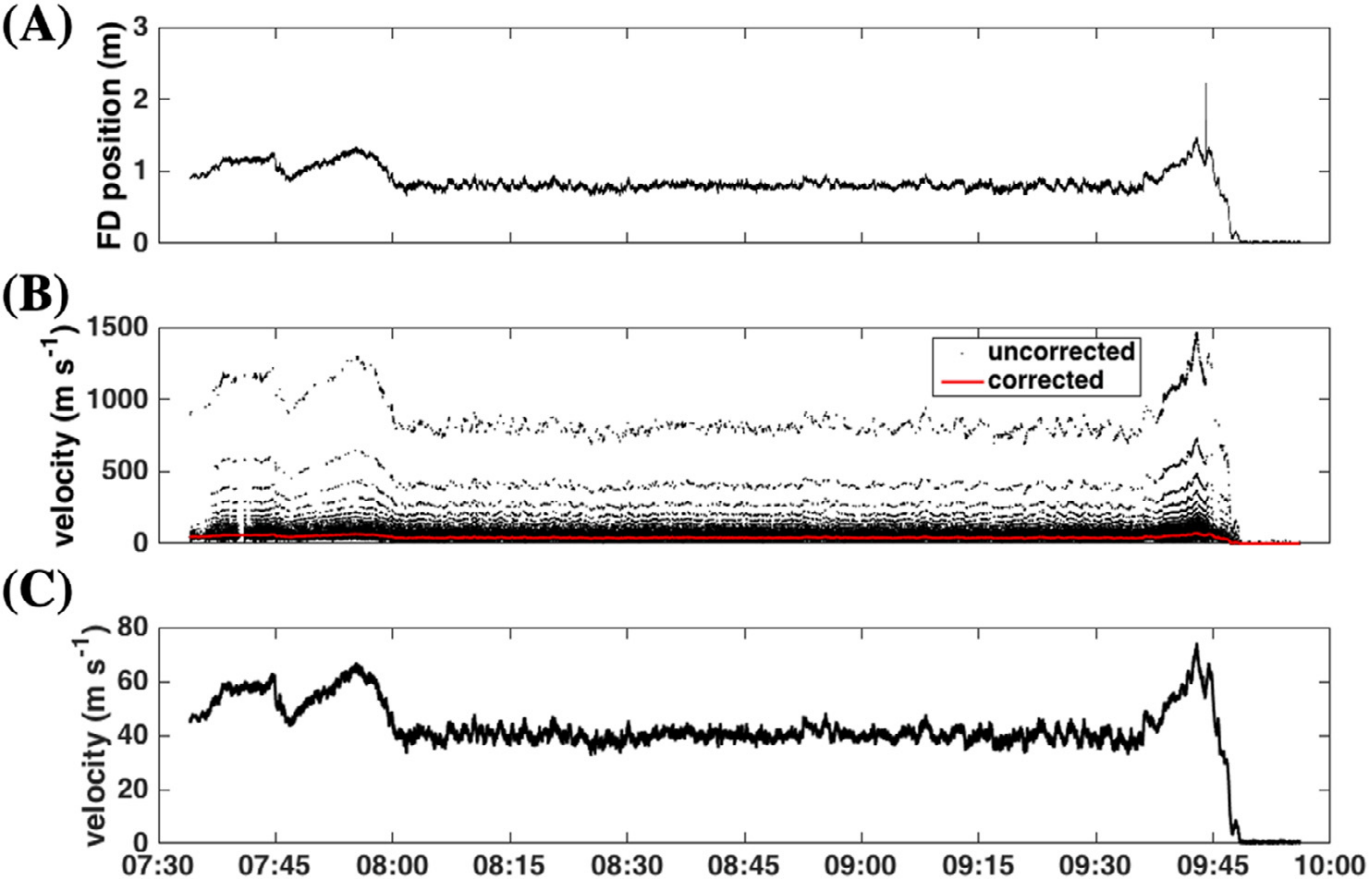
Example inertia navigation unit data from 23 May 2015. (**A)** First difference (*FD*) in position. (**B)** Velocity before (in black) and after correcting the time base (in red). (**C)** Velocity after correcting the time base.

INU data were post-processed to correct for missing data points and irregularities in the timebase. Dropped observations were identified from spikes in the velocity time series (Fig. 6A) and were filled by linear interpolation of the surrounding data. For example, Fig. 6A shows a dropped data point at ∼09:45. Next, timebase irregularities were corrected to a uniform timebase. Fig 6B shows velocity before the timebase correction (Fig. 6C). The corrected timebase assumes a uniform data rate between the first and last recorded data points and agrees with the instrument output frequency of 50 Hz.

**Fig. 7:**
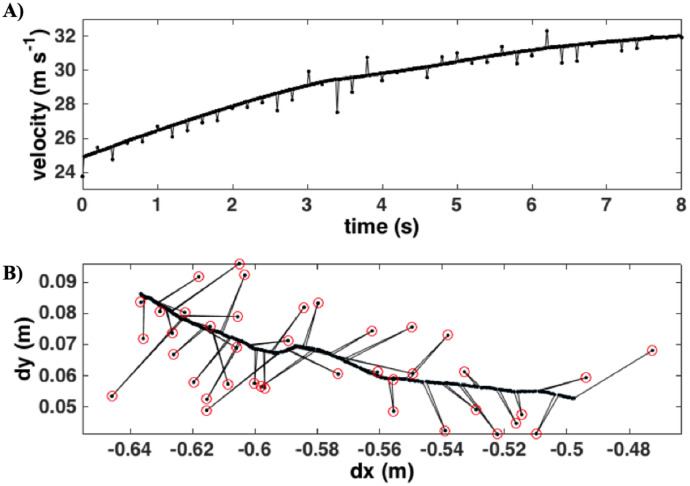
**(A)** Velocity after correcting the time base for an eight-second data segment. (**B)** The first difference in position components (*dx*) for the data in panel A. Position shifts are identified by the red circles and align with velocity spikes in panel A.

Occasional small spikes in velocity remained after the timebase correction (Fig. 7A). The spikes generally occurred every ten observations (0.2 s apart) and are small sudden shifts in the INU position likely due to the receipt of newly acquired GPS fixes. Fig. 7B shows the first difference in position (from the data shown in panel a) and illustrates the relatively small magnitudes of the shifts, 0.0276 ± 0.0256 m, for all the INU data analyzed. INU orientation data (yaw, pitch, and roll) were filtered with a five-point (0.1 s) median filter to reduce noise and then nearest-neighbor averaged.

**Fig. 8:**
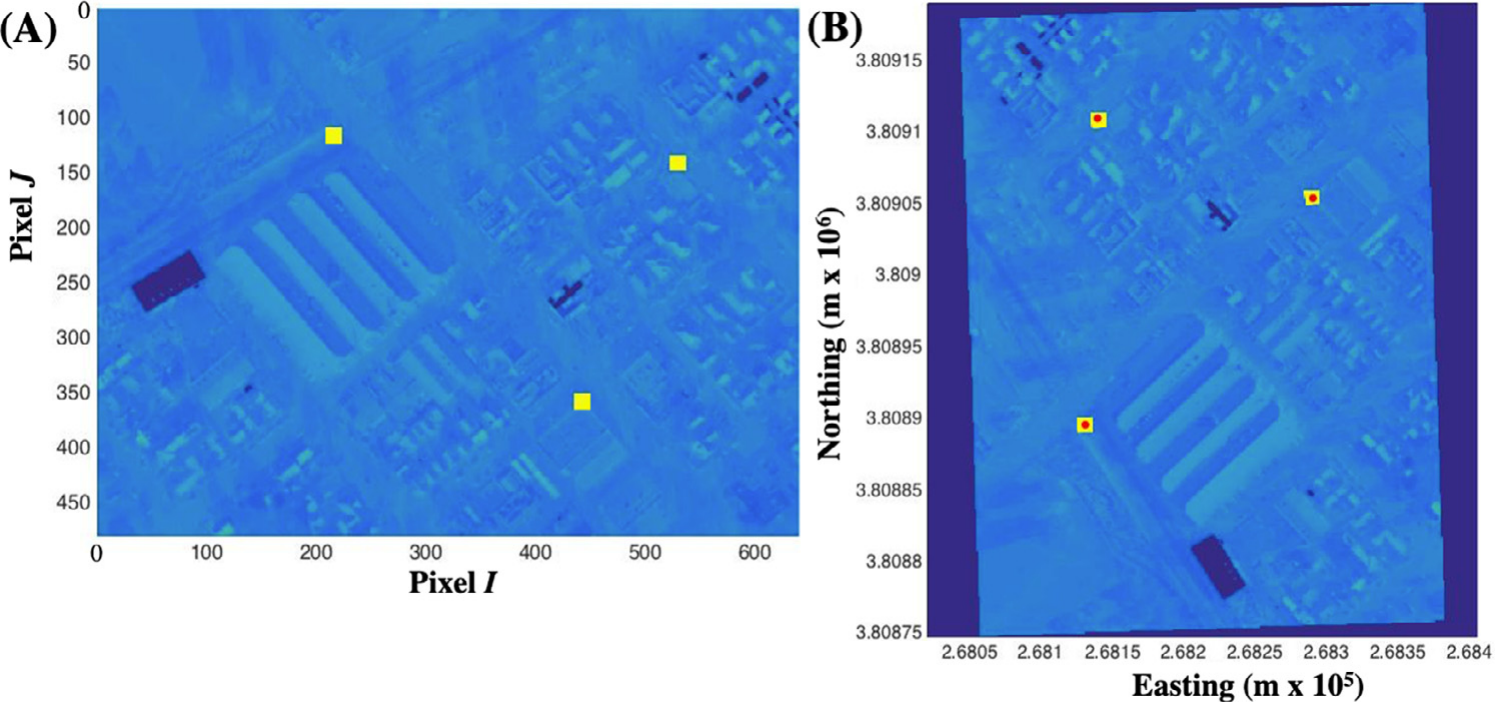
**(A)** Thermal infrared camera image. Yellow pixels were used to determine camera sensor size. I and J are image pixel coordinates. (**B)** Optimized pixel location based on GPS coordinates of reference pixels.

The first image geo-registration step was to define each camera's Field of View (FOV), determined by a ground reference method. The method involved determining each camera's true sensor size, given that all other parameters relevant to the FOV are known (lens focal length, airplane altitude above ground). First, three scene landmarks were identified in the raw TIR image (640 × 480 pixels) of a city scene (buildings and roads) (Fig. 8A). This allowed determination of the camera sensor's size. The center pixels’ longitude and latitude for the features (I_n_, J_n_) were determined from Google Earth, where *n* is the pixel number and ranges from 1 to 3.

Next, the image projected onto a UTM (coordinated universal time) grid and the alignment between the three ground reference pixels and their corresponding coordinates are optimized using ImGRAFT. Fig. 8B shows the optimized projection for aligning the three reference pixels (yellow) with related GPS locations (red dots). Several camera parameters in ImGRAFT were varied during optimization, including camera sensor size, translation (*x, y*), and *yaw*. Initially, temporal and angular offsets between each camera and the INU are unknown; therefore, allowing small perturbations to the camera's position and heading was necessary for proper alignment based on optimization. Several parameters were fixed. The camera height above ground was set, the camera pitch was assumed 90° (nadir view), and the camera roll was assumed 0° These parameters all were consistent with the airplane flying straight and level during this image acquisition. Based on the best ground reference point alignment, the true sensor sizes for the A655s and 7 K HDPro cameras were 11.69 × 8.80 cm and 23.43 × 15.00 cm, respectively.

The projection of the video image required several assumptions. First, the camera-INU alignment was assumed perfect – i.e., angular offsets (*yaw, γ, pitch, δ*, and *roll, σ*) between cameras and the INU were 0° The INU and cameras also are assumed to be at the airplane's center of rotation. Second, cameras were assumed to be mounted in perfect nadir orientation (downward-facing for level flying). For each image time, *latitude*+*Δlat, longitude*+*Δlon*, airplane altitude, *Z*, and *yaw, γ*, were linearly interpolated from the INU data and passed to ImGRAFT, with *pitch, δ,* set to 90° (nadir), and *roll, σ*, set to 0° *Δlat* and *Δlon* account for spatial corrections of image geolocation from the INU γ, *δ*, and *σ* and are described in Eqs. (1)–(6):

(1) $$\begin{array}{ccc} \Delta \mathrm{la}t_{1} & = & |Z*\text{tan}\left(\sigma \right)|*\text{sin}\left(\gamma -90\right)\;\text{for}\;\sigma >=0\;\text{or}\\ \Delta \mathrm{la}t_{1} & = & |Z*\text{tan}\left(\sigma \right)|*\text{sin}\left(\gamma +90\right)\;\text{for}\;\sigma <0 \end{array}$$

(2) $$\begin{array}{ccc} \Delta \mathrm{la}t_{2} & = & |Z*\text{tan}\left(\delta \right)|*\text{sin}\left(\gamma \right)\;\text{for}\;\delta >=0\;\text{or}\\ \Delta \mathrm{la}t_{2} & = & |Z*\text{tan}\left(\delta \right)|*\text{sin}\left(\gamma -180\right)\;\text{for}\;\delta <0 \end{array}$$

(3) $$\begin{array}{ccc} \Delta \mathrm{lo}n_{1} & = & |Z*\text{tan}\left(\sigma \right)|*\text{cos}\left(\gamma -90\right)\;\text{for}\;\sigma >=0\;\text{or}\\ \Delta \mathrm{lo}n_{1} & = & |Z*\text{tan}\left(\sigma \right))|*\text{cos}\left(\gamma +90\right)\;\text{for}\;\sigma <0 \end{array}$$

(4) $$\begin{array}{ccc} \Delta \mathrm{lo}n_{2} & = & |Z*\text{tan}\left(\delta \right)|*\text{cos}\left(\gamma \right)\;\text{for}\;\delta >=0\;\text{or}\\ \Delta \mathrm{lo}n_{2} & = & |Z*\text{tan}\left(\delta \right)|*\text{cos}\left(\gamma -180\right)\;\text{for}\;\delta <0 \end{array}$$

(5) $$\Delta lat=\Delta lat_{1}+\Delta lat_{2}$$

(6) $$\Delta lon=\Delta lon_{1}+\Delta lon_{2}$$

**Fig. 9:**
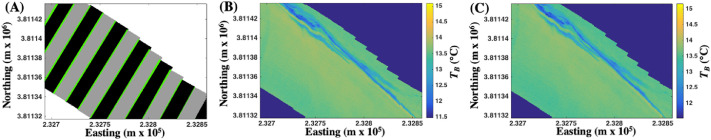
Mosaicked video image series from the thermal infrared camera, projected onto a 0.2 m resolution grid. (**A)** Projection locations of 14 images with overlapping images shown in green. (**B)** Without the INU - A655sc camera temporal offset correction and sensor size correction. (**C)** With the INU - A655sc camera temporal offset correction and sensor size correction.

Fig. 9A and B show the projection location and projection of 14 images from the TIR camera. The central 131 pixels from each image (e.g., pixel I for 175 to 305 in Fig. 8A) are used in the projection so that successive images overlap slightly (shown in green). The central region was chosen because the glare was minimal, and rectification errors were smallest towards image centers. Spatial structures associated with the oil slicks largely match, although discontinuities of up to several pixels can occur. These jumps provide empirical evidence of imperfect orientation and temporal alignment between the INU and the TIR camera.

**Fig. 10:**
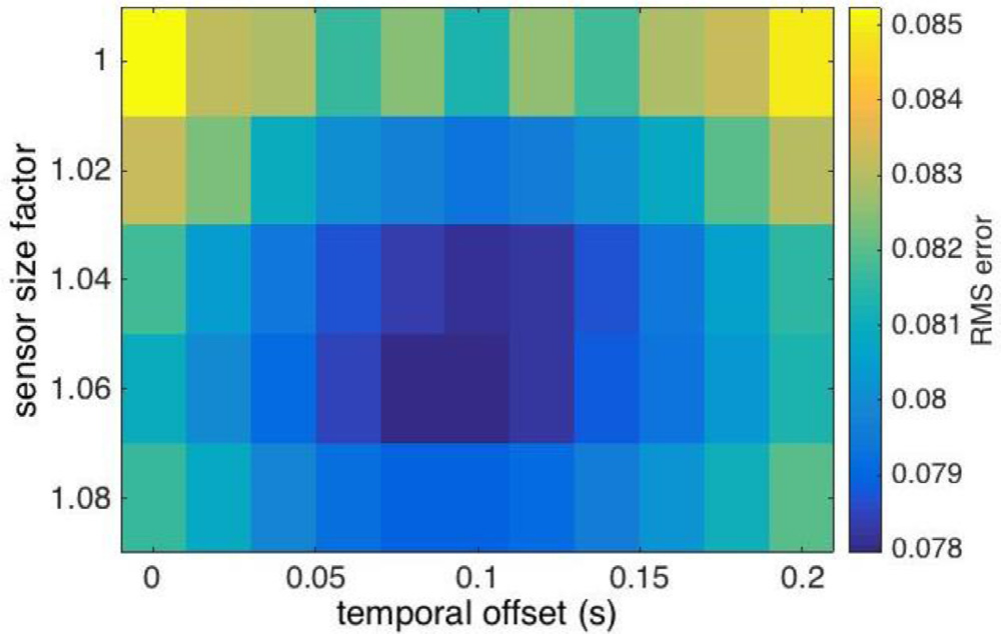
Root mean square (RMS) error between overlapping images (Fig. 9**A**) versus INU-camera temporal offset and adjustment factor to the camera's sensor size for the A655sc TIR camera.

Temporal misalignment arises from imprecision in the video time concerning frame acquisition, manifesting as a temporal offset from the INU. This offset was derived by minimizing and calculating the RMS (root mean square) difference for overlapping pixels between adjacent images of a projection of multiple overlapping images onto a 0.2 m resolution grid difference. Additionally, the RMS difference also was minimized for camera sensor size. Fig. 10 shows the average RMS error for the TIR camera for temporal offsets ranging from 0 to 0.2 s (x-axis) and a correction on the sensor size ranging from 100 to 108% (y-axis). The average RMS error was a minimum for a 0.1 s temporal offset and a sensor size factor of 106%. Application to the 7 K HDPro video camera yielded a temporal offset of approximately 0.1 s and a sensor size factor of 106%.

These additional corrections reduced geolocation errors. Photo mosaicking defects (discontinuities) at the image edges generally were less than one pixel but could be up to several pixels. Additional corrections could improve georectification, such as deriving orientation offsets between the INU and the cameras. However, this level of georectification accuracy was judged sufficiently accurate for the present application. Specifically, errors associated with inaccurate geo-rectification only introduce error through double pixel counting between frames. Pixel size variations from orientation errors are a tiny fraction of the pixel size. The vast majority of pixels are not double-counted – which only occurs near the image subset edges. This also is true because the visible imagery is not used to assess oil thickness but rather to segregate oil-related thermal structures from those unrelated to oil. Thus, mismatches between the orientation of the two cameras do not change the amount of oil, only the efficiency of the thermal analysis routines.

**Fig. 11:**
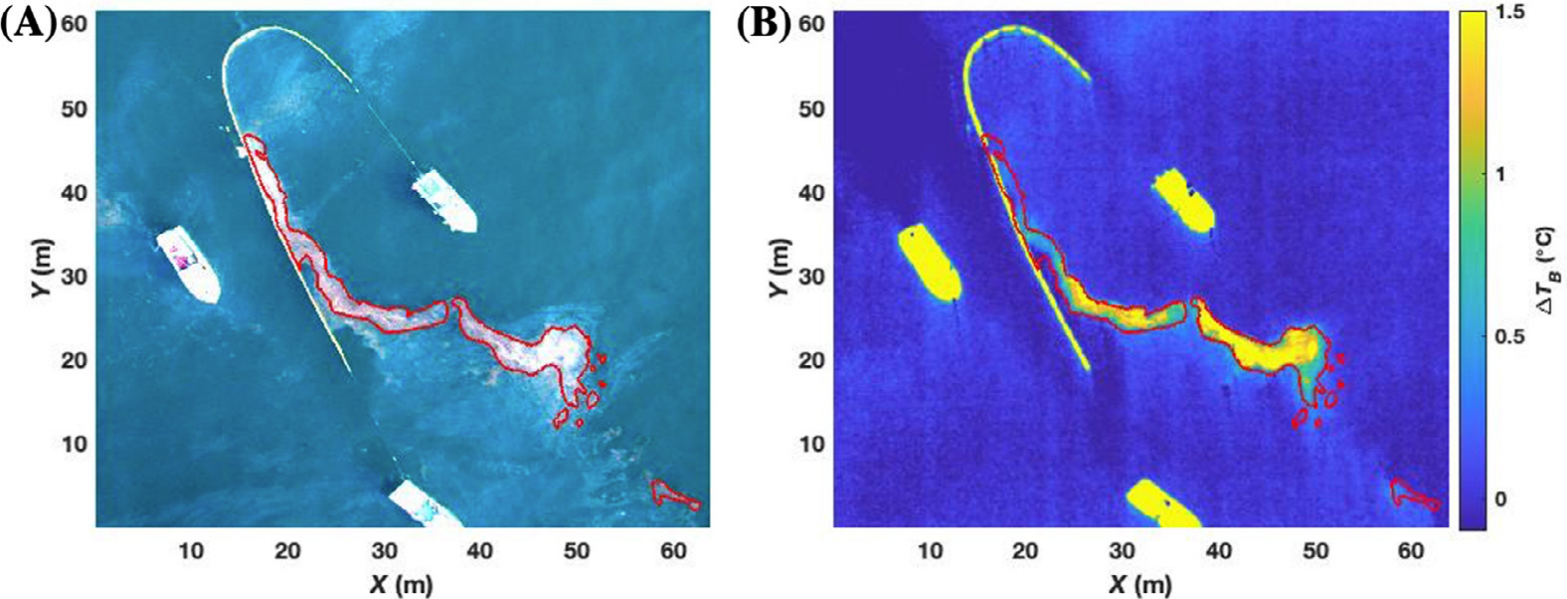
**(A)** Visible image of an oil collection on 23 May 2016, and the semi-supervised thick oil classification outlined in red**. (B).** Thermal image brightness contrast (*ΔT_B_*) overlaid on the same grid as the visible image using the Digital Elevation Model.

Co-registration of the TIR and VIS imagery for the collects involved a Digital Elevation Model (DEM) and a camera model, which projects the TIR image onto the VIS image's DEM grid [32]. The DEM is a gridded coordinate system with known elevations on which the image is projected. For our application, the visible image's gridded coordinate system (in pixels) provided the DEM with an altitude of zero for the sea surface. Specifically, the initial camera model assumed a downward-looking TIR camera (pitch set to 90°), with roll and yaw set to 0. The TIR camera location (*x, y, z*) was set to (0, 0, *Z*), where *Z* is the altitude. To optimize the cross-registration overlay, the camera model required “ground-truthing” control points – specifically, three distinct locations in a triangular (non-linear) orientation that define a plane. The control points were selected by manually matching oil features and patterns (Fig. 11). The control points are used to best estimate the exact spatial location in image pairs, preferentially encompassing the area of interest (the profile). Control points are spaced widely to optimize overlay accuracy. A good selection of control points is validated by comparing TIR contours with VIS imagery structures (Fig. 11).

Next, ImGraft optimizes the initial camera parameters to determine the best alignment of the image control points over the visible DEM control points. Camera parameters that were varied include the TIR camera's relative location to the DEM grid, the TIR camera view orientation (yaw, pitch, roll), and the TIR camera focal length (in pixel units). Parameters not allowed to vary included the world coordinates of the TIR camera (both TIR and VIS cameras were fixed in space relative to each other) and the radial and tangential distortion coefficients, as co-alignment near the images’ edges were unimportant due to photo mosaicking only using the image's central portions. The toolbox projects the TIR image onto the VIS image coordinate system (the DEM) using a two-dimensional gridded interpolation and the optimized camera model parameters.

### Pixel classification

Pixel classification was primarily by *T_B_*, except for pixels where *T_B_* was near zero - a situation that arises for thin oil due to emissivity wherein solar heating matches the apparent cooling from emissivity. These pixels are challenging to classify as oil with *T_B_*=0 cannot be distinguished from oil-free seawater based solely on *T_B_*. These pixels were addressed by a semi-supervised analysis that included visual inspection of the VIS imagery. Specifically, a mask image was created by tracing out artifacts within the visible image in the ImageJ program. ImageJ is described in Schneider et al. [33]. Then, the mask was brought into MATLAB to replace all artifact pixels within the TIR image to NaN values so that these pixels would not affect any of the filtering processes. The TIR image was de-speckled using the imclose.m function utilizing a 5 × 5-pixel structural element, and smoothing was applied using the smooth.m function.

Thick oil pixels were classified based on *T_B_* and the texture of the oil. A contrast-enhanced version of the visible image differentiated thin oil and water pixels. For context, the water aft of the boat's stern (where you would expect oil-free water) provided the reference for the true color of oil-free water.

### Sea surface water and oil temperatures

#### Brightness temperature contrast: collects

**Fig. 12:**
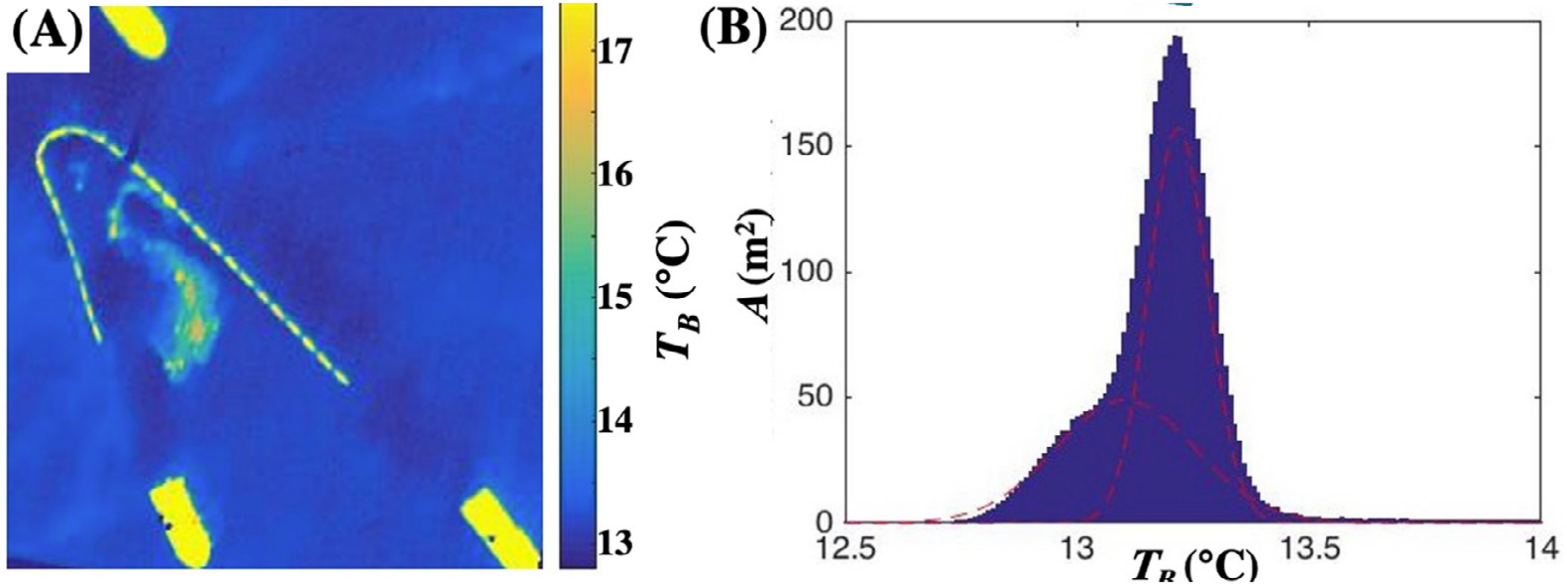
(**A)** Collect scene. (**B)** Sea surface brightness temperature (*T_B_*) probability distribution (Φ) for the image in panel A. Also shown are Gaussian fits to water (*Φ_W_* and *Φ_W1_*) and oil (*Φ_o_*). Data key on the figure.

**Fig. 13:**
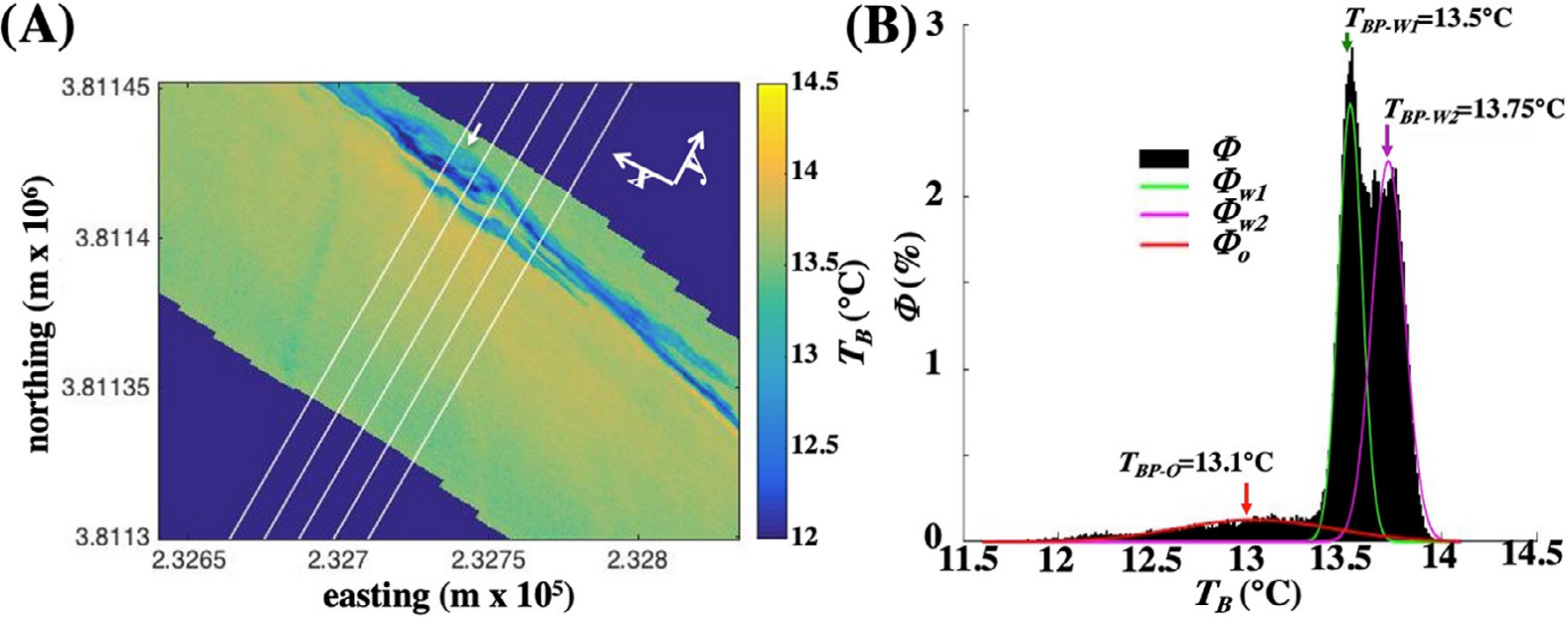
(**A)** Slicks survey image, (**B)** Sea surface brightness temperature (*T_B_*) probability distribution (Φ) for the image in panel A. Also shown are Gaussian fits to water (*Φ_W1_* and *Φ_W2_*) and oil (*Φ_O_*). Data key on the figure. Image acquired 23 May 2016.

The oil Δ*T_B_* is relative to the water brightness temperature, *T_BW_*. For the collects, *T_BW_* is derived from the probability distribution of *T_B_*, Φ(*T_B_*), which is analyzed to identify the different scene elements (Fig. 12). Specifically, the oil temperature probability distribution was modeled by a broad distribution whereas the two narrower distributions modeled the oil-free water and wake,

(7) $$\Phi =\Phi_{o}+\Phi_{w}+\Phi_{w1}=a_{o}\;e^{-\frac{{\left(T_{BP\_o}-T_{B}\right)}^{2}}{\sigma_{o}^{2}}}+a_{w}e^{-\frac{{\left(T_{BP\_w}-T_{B}\right)}^{2}}{\sigma_{w}^{2}}}+a_{w1}e^{-\frac{{\left(T_{BP\_w1}-T_{B}\right)}^{2}}{\sigma_{w1}^{2}}}$$

where *a_o_, a_w_*, and a_w1_ are the amplitudes or maxima of the distributions, $\Phi_{o}$,$\;\Phi_{w}$, and $\Phi_{w1}$, for the oil, water, and wake masses, respectively, with distribution half-widths, *σ_o_, σ_W_*, and *σ_W1_* and peaks at$T_{BP\_o}$, $T_{BP\_w}$, and $T_{BP\_w1}$. *Φ_O_* is significantly broader than for water, *Φ_W_*, which is expected given that *h* spans orders of magnitude and the functional dependency of *h*(Δ*T_B_*). In Fig. 13, *Φ_o_* is cooler than the water Gaussian functions, *Φ_w_* and *Φ_w1_*, 13.1 °C for oil versus 13.5 °C and 13.75 °C for the wake and for seawater. Thus, *T_B_* for thin oil is less than for water because *ε* is lower for oil than seawater.

#### Brightness temperature contrast: slick surveys

Before modeling the sea surface cross-slick *T_B_* profile, outliers were removed in the cross-slick temperature profile, *T_BW_*(*y*). Water pixels were identified as those for which *T_B_* lies within the range where *Φ_W_* > 0.01*a_W_* is classified as water. Outliers were defined based on *σ* derived from *Φ_W_(T_B_*) from (Eq. (7), Fig. 13). Where there were two water masses on either side of the slick, Φ was modeled by two Gaussian functions and *σ* = (*σ_W_* + *σ_W1_*)/2. A 1-m moving average of *T_BW_*(*y*) was used to identify further outliers. Specifically, outliers are defined as more than 2*σ* from the moving average and are interpolated.

*T_BW_*(*y*) is determined from a combination of a linear polynomial and a sinusoidal function fit to non-outlier water pixels,

(8) $$T_{BW}\left(y\right)=b+cy+\sin\left(dy\right),$$

where *b, c*, and *d* are fit parameters. *T_BW_*(*y*) is a two-part piecewise linear function with a transition gaps from $y_{1}$ to $y_{2}$, i.e., across the oil slick where there is a discontinuity. A Gaussian transition function gap-fills,

(9) $$f\left(y\right)=f_{1}\left(y\right)\left(1-e^{-\frac{{\left(y_{1}-y\right)}^{2}}{{\left(y_{1}-y_{2}\right)}^{2}}}\right)+\;f_{2}\left(y\right)e^{-\frac{{\left(y_{2}-y\right)}^{2}}{{\left(y_{1}-y_{2}\right)}^{2}}}$$

using two functions, *f_1_* and *f_2_*. The transition function is smooth through the second derivative. Evaluation of *T_BW_*(*y*) from *y*_1_ to *y*_2_ provides *T_BW_*(*y*) for the oil slicks pixels, i.e., *T_BW_* if there was no oil. *T_BW_*(*y*) is evaluated for each image strip centered at the along-slick location, *x,* i.e.*, T_BW_*(*x,y*). The brightness temperature contrast profile, Δ*T_B_*(*y*), relative to the water, is

(10) $$\Delta T_{B}\left(x,y\right)=T_{B}\left(x,y\right)-T_{BW}\left(x,y\right),$$

for *y*_1_ < *y* < *y*_2_, $\Delta T_{B}\left(x,y\right)$ is calculated by assuming a linear trend with *x* between image subsets. Given the narrow width of the image subsets (50 m, 250 pixels), non-linearity errors are small. Additionally, adjacent image subsets have ∼90% overlap. Overlapping $\Delta T_{B}\left(x,\;y\right)$ values between adjacent image subsets are averaged.

**Fig. 14:**
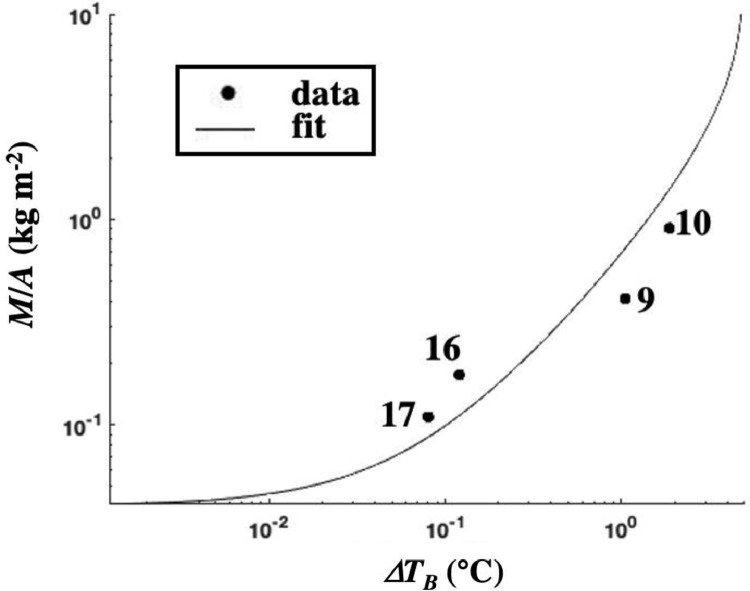
Brightness temperature contrast, *ΔT_B_*, versus the mass, *M*, to area, *A*, ratio, for calibration "collects" and asymptotic exponential fit. Data key on figure. From Leifer et al. [1].

Based on the approach described in this manuscript, a calibration function was developed based on four collects with two additional constraints. Specifically, in the thick-oil limit, there is no additional energy to be absorbed, i.e., the function asymptotes, and in the zero-oil limit (*h* = 0), *ΔT_B_* = 0. Details on the collects and the empirical model are provided in Leifer et al. [1]. In brief, the model is iterative and relates the collected oil mass, *M,* to the brightness temperature area, $A(\Delta T_{B})$, by:

(10) $$M\left(\Delta T_{BO}\right)=k\left(\Delta T_{BO}\right)\int_{0}^{\infty }1*A\left(\Delta T_{BO}\right)d\Delta T_{BO}+N$$

(11) $$M\left(\Delta T_{BO}\right)=k_{2}\left(\Delta T_{BO}\right)\int_{0}^{\infty }k\left(\Delta T_{BO}\right)A\left(\Delta T_{BO}\right)d\Delta T_{BO}+N$$

where *k* is the initial scaling parameter (set to unity) and *N* is the error. Then, *k_2_* is calculated based on $M(\Delta T_{BO})$ from all the collects by minimizing *N*. Fig. 14 shows the calibration function. Variation between the model and measured *M/A* arises from different environmental conditions between collects - successful collects were challenging with only one to two successful collects per day.

The approach was applied to two slicks on 23 May and 25 May as described in Leifer et al. [1] and found 310 and 2670 kg, for surveys of 2.2 km versus 1.1 km, with respective uncertainties of 25% and 7%.

Uncertainty was by 10,000 Monte Carlo simulations with uncertainty in *T_B_* used in the *ΔT_B_* calculation based on the standard deviation of the non-oil sea surface temperature (0.084 K). This value was taken as the uncertainty in each collect measurements that underlay the calibration function. Each Monte Carlo simulation calculated a new calibration function which then was applied to the slick data, with the probability distribution function of the resultant floating oil mass fit by a Gaussian function with a half-width that defined the uncertainty.

## Discussion and conclusion

The lack of quantitative field validation remains a key factor impeding the further use of remote sensing in oil spill response. The Seep Assessment Study approach uses an in-scene calibration of oil thickness versus thermal contrast remote sensing for thick floating oil. In essence, the Seep Assessment Study is a reverse planned oil release experiment. In a planned-release experiment, a known amount of oil is released and the remote sensing attempts to account for all the oil. Planned releases have not been legal in US waters since 1990 [34] and are not allowed in the water of most other countries. Results from planned release experiments have found poor agreement [35,36] or only investigated detection [37].

In the SAS experiment, the Coal Oil Point seep field perennial natural thick oil slicks were studied with no limit to the number of repetitions that could be attempted. In fact, many collects were unsuccessful even after the team learned how to conduct the experiment, with numerous failures in the early attempts (there were several initial trial days with no successes). In the reported planned release experiments, there are typically only a few releases. These advantages demonstrate the value of the Coal Oil Point seep field for seep science.

There are many areas for further development. The thermal brightness contrast depends on several oil parameters, environmental characteristics, and scene factors such as the air-water temperature difference, the oil characteristics, solar insolation, etc. The in-scene calibration accounts for these factors – the oil slick and the slick calibration are for the same conditions and the same oil. However, in many conditions, such as during an oil spill, in-scene calibration is infeasible.

Thus, developing a theoretical framework is critical to extending one set of field observations to another spill. Specifically, a numerical heat model that includes radiative and heat transfer processes, including turbulence heat transfer, is needed. Numerical models require validation by comparison with the vertical thermal structure of oil slicks of different thicknesses, density, emulsion levels, and different environmental conditions in the lab and field. Three manuscripts on these aspects are under review.

Additionally, improvements are needed to improve collects – the three boats needed to coordinate very closely. For example, an aerostat video system that provides real-time overview feedback to the captains could improve performance and shorten training time. Still, the most important factor was practice by the boat captains.

To our knowledge, no other quantitative oil slick remote sensing approach (and there are very few demonstrated approaches, even fewer using operational as opposed to research instruments) has been successfully, quantitatively field validated. This study also reports uncertainty, which is unusual for oil spill science. As such, the Seep Assessment Study methodology advances the state-of-the-art for quantitative oil thickness remote sensing significantly.

## Declaration of Competing Interest

None

The authors declare that they have no known competing financial interests or personal relationships that could have appeared to influence the work reported in this paper.

## Data Availability

Data are shared with companion paper.
